# Road traffic related mortality in Vietnam: Evidence for policy from a national sample mortality surveillance system

**DOI:** 10.1186/1471-2458-12-561

**Published:** 2012-07-27

**Authors:** Anh D Ngo, Chalapati Rao, Nguyen Phuong Hoa, Damian G Hoy, Khieu Thi Quynh Trang, Peter S Hill

**Affiliations:** 1 Social Epidemiology and Evaluation Research Group, School of Health Sciences, University of South Australia, Room P4-23, Playford Building, City East Campus, Adelaide, SA 5000, Australia (Formerly, Vietnam Evidence For Health Policy Project, School of Population Health, University of Queensland, Health Strategy and Policy Institute, 138 Giang Vo Street, Hanoi, Vietnam; 2 School of Population Health, University of Queensland, Herston Rd, Herston, Queensland 4006, Australia; 3 Hanoi Medical University, 1 Ton That Tung Street, Hanoi, Vietnam; 4 Health Environment Management Agency, Ministry of Health, 1 Nui Truc Lane, Nui Truc Street, Hanoi, Vietnam

**Keywords:** Road traffic injuries, Mortality, Helmet law, Verbal autopsy, Vietnam

## Abstract

**Background:**

Road traffic injuries (RTIs) are among the leading causes of mortality in Vietnam. However, mortality data collection systems in Vietnam in general and for RTIs in particular, remain inconsistent and incomplete. Underlying distributions of external causes and body injuries are not available from routine data collection systems or from studies till date. This paper presents characteristics, user type pattern, seasonal distribution, and causes of 1,061 deaths attributable to road crashes ascertained from a national sample mortality surveillance system in Vietnam over a two-year period (2008 and 2009).

**Methods:**

A sample mortality surveillance system was designed for Vietnam, comprising 192 communes in 16 provinces, accounting for approximately 3% of the Vietnamese population. Deaths were identified from commune level data sources, and followed up by verbal autopsy (VA) based ascertainment of cause of death. Age-standardised mortality rates from RTIs were computed. VA questionnaires were analysed in depth to derive descriptive characteristics of RTI deaths in the sample.

**Results:**

The age-standardized mortality rates from RTIs were 33.5 and 8.5 per 100,000 for males and females respectively. Majority of deaths were males (79%). Seventy three percent of all deaths were aged from 15 to 49 years and 58% were motorcycle users. As high as 80% of deaths occurred on the day of injury, 42% occurred prior to arrival at hospital, and a further 29% occurred on-site. Direct causes of death were identified for 446 deaths (42%) with head injuries being the most common cause attributable to road traffic injuries overall (79%) and to motorcycle crashes in particular (78%).

**Conclusion:**

The VA method can provide a useful data source to analyse RTI mortality. The observed considerable mortality from head injuries among motorcycle users highlights the need to evaluate current practice and effectiveness of motorcycle helmet use in Vietnam. The high number of deaths occurring on-site or prior to hospital admission indicates a need for effective pre-hospital first aid services and timely access to emergency facilities. In the absence of standardised death certification, sustained efforts are needed to strengthen mortality surveillance sites supplemented by VA to support evidence based monitoring and control of RTI mortality.

## Background

At its current stage of development, Vietnam has a blend of road users which typically consists of pedestrians, bicycles, motorcycles, trucks, minibuses, buses and cars. Rapid economic growth has been accompanied by an explosive increase in motorization in Vietnam with motorcycles often dominating the traffic flow in mixed road networks. There has been a rapid increase in the number of traffic injuries and deaths: between 2006 and 2010, the Ministry of Health (MoH) reported 15,000 to 18,000 deaths each year from RTIs [1], compared to 6,394 deaths in 1998 [2]. The average annual road traffic injury death rate of approximately 18 per 100,000 population over the past five years [1] is one of the highest rates in the Western Pacific region [3]. Males aged from 15 to 49 years constitute the majority of deaths and injuries on the road, and transport crashes are the leading cause of mortality among this age group [4]. While the poor transport infrastructure and road network continue to contribute to the traffic injuries and deaths, improved road conditions, especially on national highways, have also resulted in high speed traffic, and consequently the increased frequency of crash and severity of injury. In addition, physical road safety measures such as appropriate road lane allocation, improvement of surface conditions, paving of shoulders, and installation of traffic signs, signals and pedestrian crossings remain inadequate [5].

In attempts to reverse the situation, several policy and programmatic responses have been implemented. The most important policy initiative was the promulgation of the compulsory helmet legislation in December 2007 [6]. In addition, national road safety campaigns have targeted road safety during special occasions throughout the country when there is a high volume of traffic. These include the national traffic safety month conducted in September, and the national road safety campaign around the Lunar New Year (Tet) in January. Typically, these campaigns involve an increased presence of traffic police to foster compliance with traffic regulations and maintain order. Communication activities raise public awareness on traffic regulations and road safety measures during these campaigns [7].

Despite the magnitude of this problem, the data collection system for RTIs and mortality is inconsistent and incomplete in Vietnam. The National Traffic Safety Committee of the Ministry of Transport, and the Ministry of Police are responsible for recording and providing official data on RTIs and mortality. In addition, the MoH collects death records from commune health stations, and central and provincial hospitals. However, there is great variation among the different data sources and it is claimed that official data underestimate the true burden by at least 30% [3]. For example, one study in Thai Nguyen province found that during a five-year period (2000-2004), 1,373 non-fatal road traffic injuries were reported by the police, compared to 6,069 cases recorded in hospitals [8]. In the first six months of 2009, the MoH reported 68,510 hospitalised RTI cases while the Ministry of Transport recorded only 5,127 cases [9]. Moreover, the underlying distribution of mortality such as the mode of injury, anatomical site(s) of injury, and the primary and secondary causes of death are not available from the data collection system nor from existing studies.

Routine, reliable mortality statistics from efficient civil registration systems are the optimal source for this detailed information on mortality. However, fully functional civil registration and vital statistics systems are still to be established in many low and middle income countries, including Vietnam [10]. As an interim measure, a sample mortality surveillance system has been developed in Vietnam, to provide nationally-representative data on mortality and causes of death [11]. The system recorded deaths that had occurred in 2008 and 2009, with the probable cause(s) of death established using verbal autopsy (VA) methods[12]. This paper presents characteristics, user type pattern, and cause of 1,061 deaths attributable to RTIs, which were ascertained from the surveillance sites over this two-year period, and discusses policy implications for the prevention of road accident mortality in Vietnam.

## Methods

### Sample mortality surveillance system design

**Figure 1:**
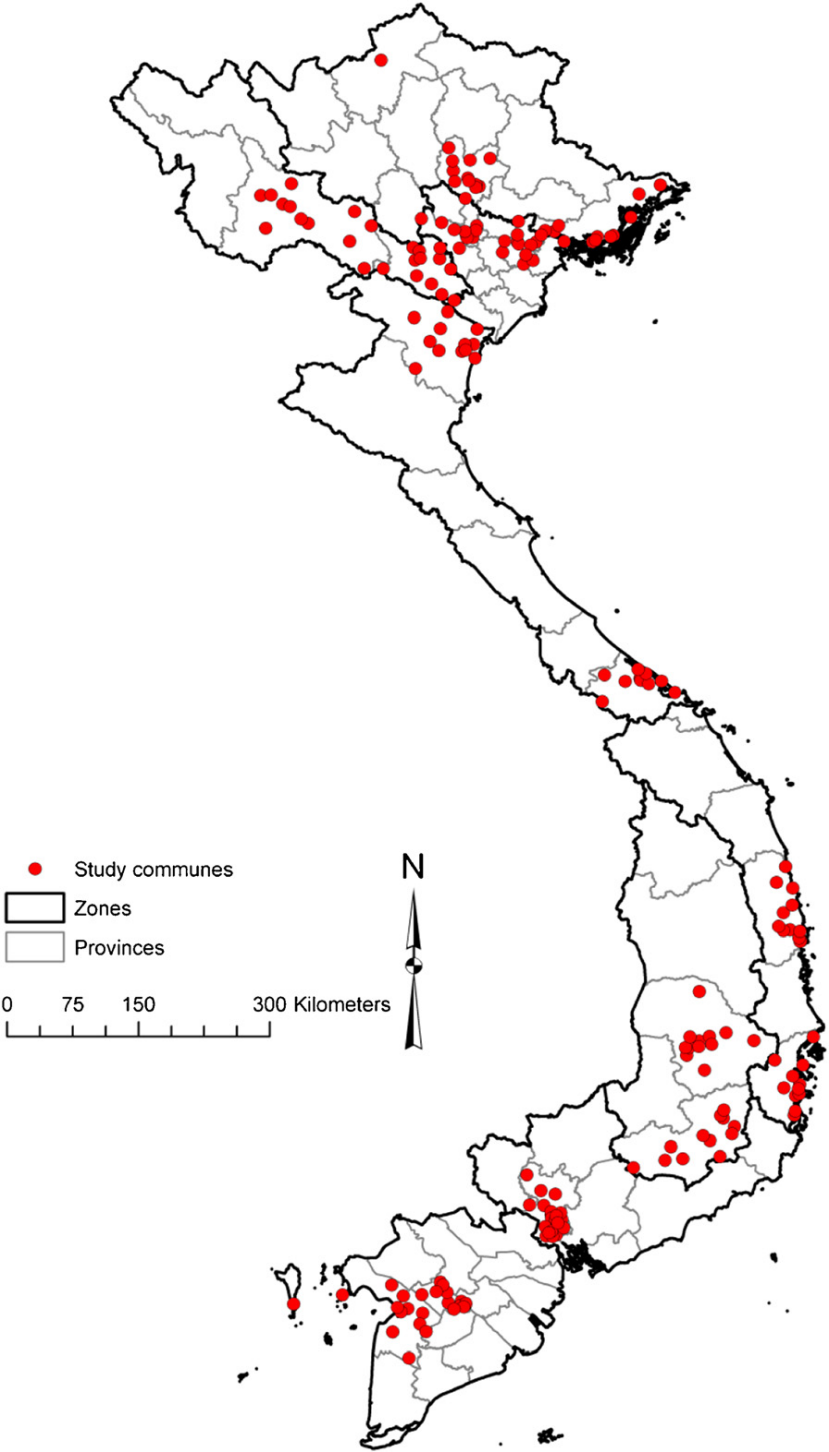
Distribution of communes in the sample mortality surveillance system in Vietnam.

Using a multi-stage cluster sampling technique, a mortality surveillance system was designed for Vietnam, with a sample of 192 communes in 16 provinces, representing six socioeconomic regions in Vietnam (Figure 1). The sample consisted of 668,142 households with an estimated 2,616,056 persons; accounting for approximately 3% of the Vietnamese population. The first stage involved the random selection of 16 provinces proportional to the size of the province population. The second stage randomly selected 12 communes from each province proportional to the number of households in each commune. The overall sample was estimated to yield approximately 11,000 deaths annually, which represents an optimal target for sample based mortality surveillance in a population with the age structure and mortality levels of Vietnam [13].

### Data collection and analyses

For each reference year (2008 and 2009), data collection was accomplished separately. At first, a list of deaths that occurred between January 1 and December 31 of each year was compiled from three sources at the commune level: 1) the Commune Health Station (CHS) record; 2) the Commune People’s Committee (Justice Department) report; and 3) the report from commune family planning promoters under the Population and Family Planning System. Individual records from the different sources were reconciled to generate a unique list of deaths in each commune during the reference period. In addition, capture-recapture methods were used to estimate the completeness of death recording. These methods account for the likelihood of events being missed by all sources [14]. The resultant estimates of completeness were used to adjust observed age-specific death rates and compute total mortality indicators for the sample population, and the detailed methods and results are described elsewhere [15].

For each identified unique death in a commune, demographic information of the deceased (age, sex, marital status, education, occupation, location of residence) and household address were extracted into a separate file to help locate the household for subsequent VA interviews. Trained paramedical staff from local CHSs conducted household VA interviews, as described in detail elsewhere [4]. In brief, the VA questionnaire used is a Vietnamese adaptation of the standard WHO questionnaires, and completed questionnaires are reviewed by trained physicians who assign causes of death according to the WHO prescribed guidelines [12]. VA has been recognised as a method to ascertain cause of death in the absence of an efficient vital registration system. The acceptability and reliability of this method has been confirmed in previous studies in Vietnam [4,16] and elsewhere [12,17]. Ethical approval was given by the Ethics Committee of the Hanoi Medical University, Vietnam.

RTI-related deaths were identified from VA questionnaire-based interviews. The questionnaire included question items to capture information on the date of death (transposed from the lunar calendar), place of death, survival time, health facility attended prior to death, and alcohol use before the accident. In addition, the questionnaire included an open section that recorded the respondent’s narratives of the circumstances and nature of injury and death (e.g., user type, body position(s) of the injury), complementing the data collected through structured questions. Trained physician reviewers applied conventional methods for assigning causes of death from VA [12] adapted to the Vietnamese context. All causes listed on the VA death certificate were coded to the International Classification of Diseases Version 10 (ICD-10). Subsequently, trained coders applied ICD rules for selection of the underlying cause of death. All variables from each death certificate, including the text and codes for all listed causes of death, were entered into a Microsoft Access database. Descriptive analyses were performed in STATA version 9.0 to produce frequency tables and figures on the distribution of variables related to deaths. In addition, population data by age and sex for the sample communes were obtained from the Vietnam General Statistics Office for the calculation of direct age-standardised mortality rates, using simple spread sheets.

## Results

**Figure 2:**
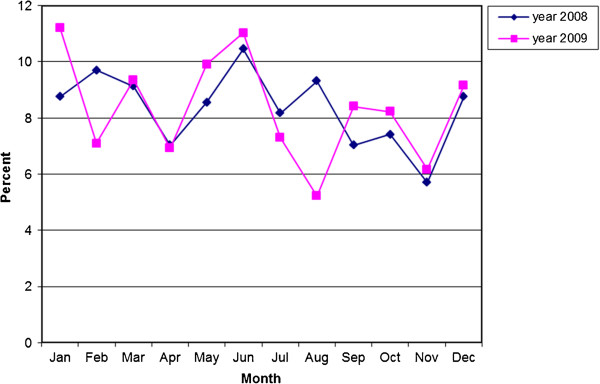
Distribution of deaths (percentage) by month during the year (lunar calendar).

There were 526 deaths from RTIs in 2008, and 535 deaths in 2009, totalling 1,061 deaths over the two-year period. The median age was 33.0 (32.5 for males and 41.0 for females). The annual crude RTI death rate was 21 (33.7 for males and 8.5 for females) per 100,000 population, and the annual age-standardised mortality rate was 20.3 (33.5 for males and 8.5 for females) per 100,000 population. The number of deaths fluctuated throughout the year, and tended to peak in June, and in December and/or January, which is the time of the Vietnamese New Year Tet festivities (Figure 2).

Table 1 shows the demographic characteristics of the deceased. A disproportionate number of deceased individuals were males (79%). When age was considered, the proportional distribution of RTI-related deaths increased from 3.68% among children below 15 years old to 11% among the 15-19 year age group, peaked at 29% among the 20-29 year age group, fell to around 16% among the 30-39 and 40-49 year age groups, before levelling off at 11% among the 50-59 year age group and the elderly. Aggregately, 73% of the deceased were aged from 15 to 49, the most economically-productive group. Despite the low median age, 65% of people who died were married. Although approximately 70% of the sample was from rural and mountainous areas, there were high proportions performing occupations other than farming (56%), and having completed primary or secondary education (81%).

**Table 1:** Demographic characteristics of the deceased

**Variable**	**n**	**%**
***Sex***		
Male	837	78.89
Female	224	21.11
***Age group***		
<15	39	3.68
15 - 19	118	11.12
20 - 29	305	28.75
30 - 39	173	16.31
40 - 49	178	16.78
50 - 59	126	11.88
60+	122	11.50
***Marital status***		
Single	376	35.44
Ever-married	685	64.56
***Occupation***		
Farmer	323	30.44
Non-farming	597	56.27
Student/children	141	13.29
***Education***		
<6 years	5	0.47
Illiterate	117	11.03
Primary school	288	27.14
Secondary school	342	32.23
High school	234	22.05
High school +	75	7.07
***Residence***		
Rural	483	45.52
Urban	322	30.35
Mountainous	256	24.12

Table 2 summarises death characteristics with regards to road user type, survival time, health facility attended prior to death, place and cause of death. Motorcycle users (either passengers or drivers) accounted for the highest percentage of deaths (58%), followed by transport users with no specified vehicle (25%), and pedestrians (11%). Deaths among car occupants and bicyclists only made up 2.5% and 3%, respectively. It should be noted that young people aged 20-29 years, mostly males, also disproportionately accounted for 35% of deaths due to motorcycle crashes. Alcohol use was reported among approximately one fifth of deaths. Almost half of the victims (45%) did not present to a health facility before death, usually because they died at the accident site or on the way to a health facility. For those that did present to a health facility, provincial hospitals were most frequently used (by 22% of victims), followed by central hospitals (12%), and then district hospitals (6%). A quarter of deaths occurred at the scene of the accident, 17% died on the way to a health facility, totalling 42% of the deaths happening before reaching a health facility; and a further 29% of people died at home. Only 29% of deaths occurred in a health facility, although some deaths at home may have followed discharge when treatment appeared unlikely to succeed.

**Table 2:** Death characteristics

**Variable**	**n**	**%**
***Road user type***		
Pedestrian	119	11.22
Cyclist	34	3.20
Motorcycle user	614	57.87
Car occupant	26	2.45
Other/unspecified	268	25.26
***Health facility attended***		
None	476	44.9
District	63	5.9
Provincial	233	22
Central	126	11.9
Other/unspecified	94	11.9
Don't know/remember	69	6.5
***Direct causes (n = 446)***		
Head	351	78.7
Multiple body regions	41	9.2
Limb	9	2.3
Unspecified	16	3.6
Other	54	8.7
***Death place***		
At home	308	29.0
In a health facility	305	28.7
On the way to a health facility	175	16.5
At the accident site	273	25.7
***Survival time***		
<1 hour	467	44.0
1 hour- <1 day	378	35.6
1- 9 days	145	13.7
10 days +	40	3.8
Don't know/remember	31	2.9
***Using alcohol before the accident***		
Yes	205	19.3
No	750	70.7
Don’t know	106	9.9

With regards to survival time, most (80%) died within the first day (ranging from 74% among pedal cyclists to 88% among car users) and 44% within the first hour (ranging from 35% among pedal cyclists to 54% among car users). Fourteen per cent died in the period from day one to nine after the accident, and 4% survived for ten days or longer before dying. When the analysis was restricted to motorcyclists, 82% died during the first day, and 48% within the first hour (Table 3). The direct cause of death was ascertained from 446 deaths (42%); of these, head injuries were responsible for 79%, followed by injuries to multiple body regions (9.2%), and injuries to limb(s) (2.3%). Injuries to other parts of the body (neck, hip, thigh, abdomen, hand) accounted for 8.7% and unspecified injuries accounted for 3.6%. It is noted that head injuries were responsible for 78% of deaths attributable to motorcycle crashes (not presented in the table).

**Table 3:** Survival time by user types

**Survival time**	**Pedestrian n(%)**	**Cyclist n(%)**	**Motorbike n(%)**	**Car n(%)**	**Other/Unspecified n(%)**
<1 hour	45(37.8)	12(35.3)	292(47.6)	14(53.8)	104(38.8)
1hour - <1 day	48(40.3)	13(38.2)	211(34.4)	9(34.6)	97(36.2)
1-9 days	19(10.0)	6(17.6)	77(12.5)	3(8.3)	40(14.9)
10 days+	6(5)	2(5.9)	23(3.8)	0	9(3.4)
Don’t remember	1(0.8)	1(2.9)	11(1.8)	0	16(6.0)

In urban areas, a higher proportion of victims were taken to provincial or central hospitals, where life-saving emergency care is available, compared to those living in mountainous and rural areas, who were more likely to present to the district hospital. Only a small fraction (4.3%) of victims in mountainous areas was able to reach a central hospital before death, compared to 13.5% and 15.5% for victims in rural and urban areas, respectively. Compared to those from rural and urban areas, the proportion of deaths occurring in a health facility was much lower for those from mountainous areas, while the proportion of victims who died on the way to a health facility was higher (Table 4).

**Table 4:** Death characteristics by residence

**Variable**	**Rural**		**Urban**		**Mountainous**	
	**n**	**%**	**n**	**%**	**n**	**%**
***Health facility***						
None	217	44.9	130	40.4	129	50.4
District	31	6.4	10	3.1	22	8.6
Province	106	21.9	79	24.5	48	18.8
Central	65	13.5	50	15.5	11	4.3
Other	40	8.3	34	10.6	20	7.8
Don’t know	24	5.0	19	5.9	26	10.2
***Survival time***						
<1 hour	215	44.5	150	46.6	102	39.8
1hour - <1 day	161	33.3	97	30.1	120	46.9
1-9 days	66	13.7	50	15.5	29	11.3
10 days+	25	5.2	10	3.1	5	2.0
Don’t remember	16	3.3	15	4.7	0	0.0
***Place of death***						
At home	127	26.3	104	32.3	77	30.1
In a health facility	149	30.8	97	30.1	59	23.0
On the way	74	15.3	39	12.1	62	24.2
At the accident site	133	27.5	82	25.5	58	22.7

## Discussion

This study is the first ever attempt to examine mortality data attributable to road traffic crashes from a national sample mortality surveillance system in Vietnam. The data collection process involved the use of surveillance to identify deaths in sampled communes during the reference period from different routine data sources, followed up by the use of household VA interviews to identify the cause for each death identified from the surveillance activity. Though the data were subject to limitations associated with the use of VA methods, the available information provides an insight into the patterns of RTIs and associated mortality. The use of multiple data sources to identify death cases allows for more complete ascertainment of deaths than hospital-based or a single routine mortality data collection system in Vietnam. The finding that only 29% of deaths occurred in a health facility further confirms that hospital-based mortality data are incomplete. While the VA method is subject to recall bias and uncertainties in determining the cause of death, detailed report of medical events or clinical presentation, and delineation of treatment is less vital to the identification of external causes of death, such as injuries, compared to other causes. Many additional details around the injury and death (e.g., road user type, circumstances leading to death, and survival time) that are valuable to cause of death coding and targeting intervention efforts can be obtained from the questionnaire-based interviews being used in the VA method [18].

The secular trend with the number of deaths reaching a peak in December and/or January (lunar calendar) around Tet national holidays is explainable given the enormously heightened vehicular road usage and increased prevalence of drink-driving during this period [19]. Also, the second peak in deaths in June coincides with university entrance examinations, and is likely to be linked to heightened road usage as large numbers of students and accompanying relatives travel to the major cities where academic centres are located. Other studies have also found summertime to be a time of increased motorcycle injuries [20,21]. Given these seasonal variations in road accident mortality, these findings are extremely relevant for planning road safety interventions in Vietnam, given the successes in limiting injury mortality during the Christmas and Easter vacation periods in Western countries [22].

The distribution of deaths varied greatly among different socio-demographic strata. Compared to other age groups, young males aged 20 to 29 dominated all deaths and deaths due to motorcycle crashes. Given that unsafe driving behaviours including speeding and drink-driving are most prevalent among this group [19], this finding is not surprising. With approximately three quarters of deaths occurring among people aged 15 to 49, the most economically-productive group, the economic loss to the family and society is high. While the proportion of the deceased who had post-high school education was almost similar to that of the national population (7.0% vs. 7.6%), the proportion who had completed secondary or high school education was higher than that of the national population (54.0% vs. 44.5%) [23]. These findings suggest there is an impact of post-high school education on the use of safety measures on the road and highlights the need to improve traffic safety education programs at the school level.

The short survival times, with high numbers of deaths occurring at the scene of the accident or before the victim reached a hospital, particularly in mountainous areas, are consistent with the severity of RTIs, but also suggest the absence or failure of first-aid and pre-hospital stabilisation of patients, with delays in response time and delayed access to life-saving trauma care. This finding is reflected in a study in Hanoi that reported more than half of the injury cases did not receive first aid at the site, and only 4% were transported to a hospital by ambulance due to the shortage of first aid and pre-hospital trauma care services at the scene, and the lack of a communication system linking the accident site with emergency service centres and health facilities [24]. Furthermore, the majority of the victims bypassed district hospitals, which may be closer compared to the provincial or central hospitals, thereby delaying access to life-saving services. It is noted that 80% deaths occurred within the first day, which is higher than in Taiwan (65%) [25] and India (72%) [26], where motorcycle deaths also account for a large proportion of the total road traffic fatalities [27,28]. One previous study indicates that up to 39% of pre-hospital deaths from road crashes can be prevented, subject to timely access to first aid or emergency care [29]. As such, many lives could have been saved if injured people were given pre-hospital trauma care and were taken to the hospital in a timely manner.

The data also provide insight into the impact of the implementation of mandatory helmet legislation, which was introduced in December 2007 [6]. Early reports suggested that the law had reduced the prevalence of head injuries among road accident patients admitted to hospital by 16% after three months [30] and the number of traffic fatalities by 1,400 cases (8%) after 12 months [31]. Yet, our data suggest that the proportion of motorcyclists in traffic fatalities (58%) remained higher than in some developing countries with similar levels of road accident mortality [32] and motorcycle use, such as Iran (12%) [33] and Taiwan (55%) [27,34]. Head injuries were the leading cause of death among motorbike users, comparable to similar statistics in countries with strictly enforced helmet legislation such as Taiwan (71.0%) [35] and Singapore (86.7%) [36]. However, these data show a different pattern compared to the US where head injuries only account for around one third of motorcycle deaths [37,38]; and helmeted motorcyclists are most likely to die from the injury to other body parts (i.e., the thorax) [38]. Though data on helmet use at the time of injury were not available from our study, observations in a sample of provinces conducted within 6 months after launching the helmet law reported average helmet wearing of 96% in motorbike users [39]. However, at the time of launching the legislation, one study found that 80% of helmets on the market failed to meet the quality standards [40]. In summary, the high rates of motorcycle fatalities and head injury-related deaths in our data signify the need to conduct further research to evaluate the effectiveness of motorcycle helmet use in Vietnam, including a qualitative assessment of the issues surrounding the enforcement of the helmet law.

As with other mortality and cause of death assessments based on sentinel surveillance systems, the data are not without limitations. Firstly, there could have been incomplete recording of deaths in the sample population clusters for the reference time period. We conducted a detailed assessment of completeness of death recording using ‘capture-recapture’ methods [14], and found that our surveillance data capture approximately 84% of the total estimated deaths in the population under surveillance [15]. Our analysis identified that there was remarkably low variation in the degree of completeness when assessed across age and sex groups, ranging from 83-86%. If we were to assume uniform under-recording of deaths by cause, then our estimated age-standardized mortality rate from RTIs would be 25 per 100,000 population; which would be 35% higher than that reported by the MoH (18.5/100,000). However, data from representative population samples may not be appropriate for assessment of mortality from RTIs, since such events may not follow a random distribution in populations as compared to other causes of death. These observations indicate the need for additional investigations into the reasons for incomplete recording, leading to initiatives for strengthening local death recording systems.

From the cause of death perspective, the validity of the VA method is dependent on the relatives’ ability to recall information and symptoms related to deaths and their willingness to report this information during the interview. At present, there are no negative cultural associations with traffic injury-related deaths that would influence the disclosure of information regarding such events [41]. Despite this advantage of the VA method pertaining to injury-related deaths, the nature of bodily injuries was identified for less than half of the deaths. Further, there is a possibility that while the deceased may have suffered multiple injuries, VA respondents may not have reported complete or accurate information to determine the specific injury. For example, deaths may have been attributed to head injury in some instances when they were actually caused by injury to other parts of the body (e.g., the thorax or abdomen). Uncertainties in the distribution of deaths by anatomical position and biological sequence of the injury, therefore, remain unresolved.

Alcohol use before the accident was reported for one fifth of deaths, which is much lower than findings from previous studies [19,42], and thus the data from this study may have been an underestimate. This may be because most respondents were not present at the scene of the accident, and were therefore unable to identify whether the deceased had used alcohol or not. Also, there is a likelihood of respondents suppressing information on alcohol consumption in relation to RTI mortality during VA interviews, due to the concern over stigma. These aspects require additional qualitative research to understand cultural sensitivities in specific VA interview situations. Furthermore, the type of vehicle was unspecified for a quarter of deaths, so the distribution of deaths by user type was not entirely accurate. Moreover, because the data described in this study refer to the date and place of death (and not of the accident), it was impossible to explore the exact seasonal distribution of crashes. Having said that, given 80% of deaths occurred in the first day after the accident, seasonal distribution of deaths provides some indication of the seasonal pattern of crashes over the year. Finally, no information on the time intervals between the accident and the presence of first aid or emergency services was recorded, so responsiveness and quality of pre-hospital trauma care cannot be evaluated.

Our initial VA methods required the interviewers to enquire and record most of the above additional descriptive information on the nature of injury and circumstances of the event within the free text narrative for such deaths. As a result of the above data shortcomings, questionnaires and interviewing protocols should be modified to capture these important variables in a thorough and systematic manner, to support future analysis of the RTI mortality in Vietnam. For example, to obtain more complete data road user types, direct closed-ended questions should be used during the interview so that more respondents will be able to recall and report the type of vehicle involved in the accident. The questionnaire could also be improved by allocating specific space to record the anatomical sites of injuries suffered by the victim, as well as to record helmet use among motorcycle users. However, the revised questionnaire should be first pilot tested for cultural appropriateness before being used to collect data.

## Conclusion

Despite the above limitations, the study clearly draws attention to several key policy and program implications. First, the absence of dependable death certification [43], particularly in a socio-cultural context where the majority of deaths do not occur in health facilities, means that policy makers are reliant on data of limited quality, which is at times contradictory, or at best, not representative. The uncertainty that surrounds critical areas of information can undermine much policy action. Positive policy initiatives such as mandatory helmet legislation need reliable and consistent data to support ongoing monitoring and enforcement of such initiatives. Projected reforms in the Health Information System need to address standardised death certification, but given the current context, need the complementary input that consistent sentinel surveillance can offer.

Second, the evidence presented in this study, combined with concerns already raised around the quality of available helmets, indicates the need for more detailed research into the phenomenon of head injury mortality among motorcyclists. Such research studies could take the form of a series of case studies that examine personal attributes such as helmet use, alcohol consumption and driving experience / skills, but also in addition examine the mechanical aspects of the accident (e.g., the quality of the motorcycle used), including an analysis of speed, road characteristics, and other factors associated with collisions. Public education to encourage the use of road safety measures, avoid irresponsible and dangerous road using behaviours, and promote helmet quality and correct application of helmets among motorcycle users is urgently needed. Third, while the enforcement of preventive measures is a critical first step to ensure the compliance with proscriptive regulation, to reduce deaths and injuries on local roads it is vital to empower and build the capacity of local communities, so that they take active roles in identifying and addressing local problems leading to road traffic collisions.

And fourth, the high proportion of deaths prior to presentation at a hospital and the pattern of bypassing district hospitals raise questions around the adequacy of first aid responses. This indicates the limited availability of pre-hospital care such as ambulance and acute care paramedics. Further, this supports popular perceptions that district hospitals are poorly staffed and resourced for dealing with severe trauma. What is now necessary is the establishment of a comprehensive pre-hospital trauma care system including the development of a cadre of acute care paramedics, and a network of strategically located ambulance retrieval vehicles to ensure provision of first aid such as maintenance of airways and control of haemorrhage, and timely access to hospital life-saving care. Efforts are also required to establish the capacity of district hospitals located close to the fatality sites in dealing with road traffic related injury. All these efforts should be made in conjunction with whole of government strategies that focus on improving the quality control process of vehicle registration and driver licensing to ensure the vehicles used meet the quality standards and to prevent unsafe use of vehicles, as well as maintenance of roads and correction of dangerous hazards. The findings from this study serve as primary evidence to support planned road traffic accident control activities in Vietnam.

## Abbreviations

RTI, Road Traffic Injury; MoH, Ministry of Health.

## Competing interests

The authors declare that they have no competing interests.

## Authors’ contributions

AN conceived research ideas, performed data analysis and drafted the manuscript with input from all other authors. All authors reviewed and approved the final version.

## Pre-publication history

The pre-publication history for this paper can be accessed here:

http://www.biomedcentral.com/1471-2458/12/561/prepub
